# Supplementary figures and images for: Safety and efficacy of bempedoic acid among patients with statin intolerance and those without: A meta-analysis and a systematic randomized controlled trial review
Source: PLoS One. 2024 Jan 26;19(1):e0297854. doi: 10.1371/journal.pone.0297854 (PMC10817114; doi:10.1371/journal.pone.0297854)

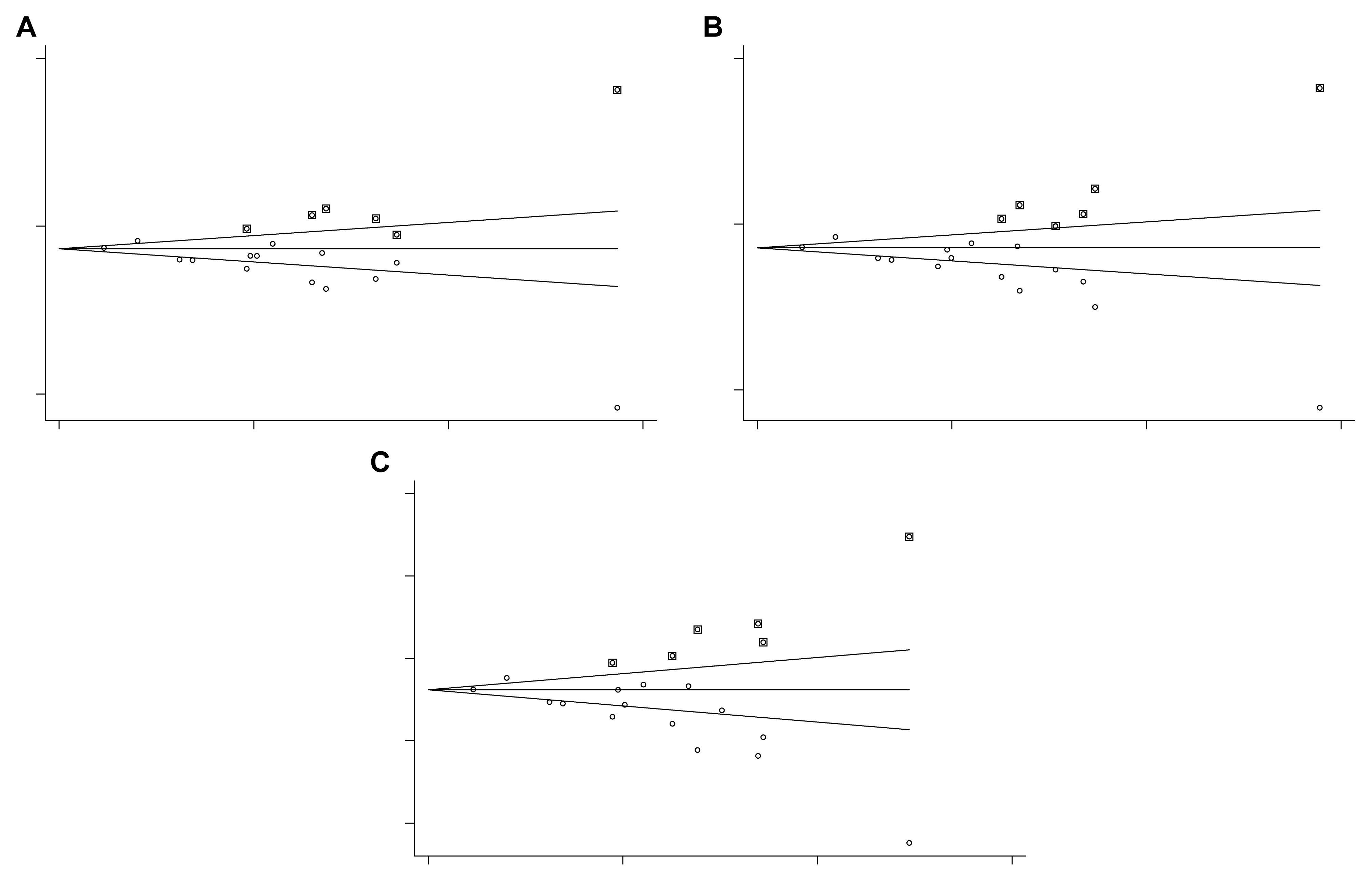

Supplement: S1 Fig — "trim and fill" technique of apolipoprotein B(A),non-high-density lipoprotein cholesterol(B) and total cholesterol(C). (TIF) [file pone.0297854.s002.tif]

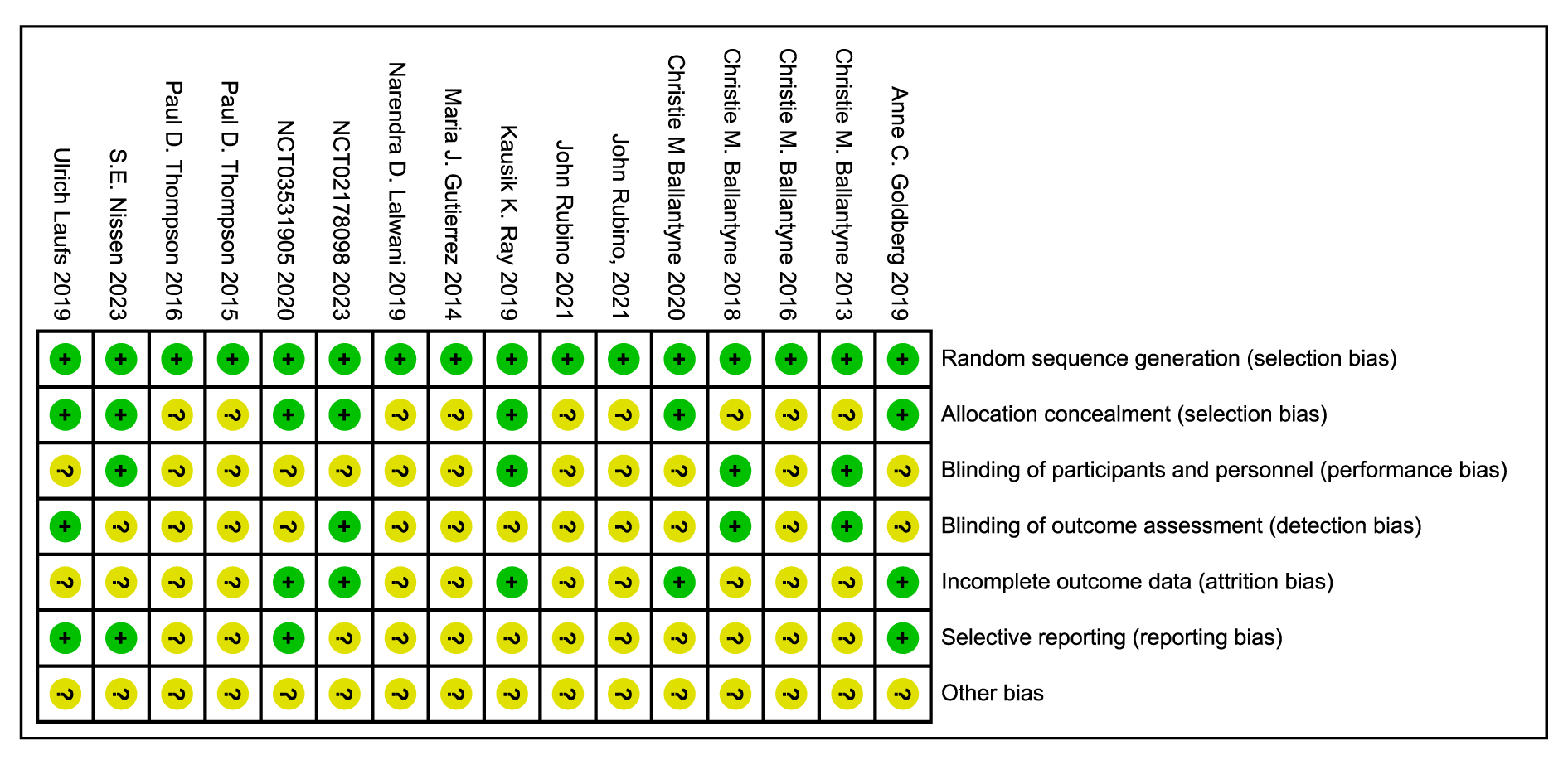

Supplement: S2 Fig — (TIF) [file pone.0297854.s003.tif]
