# Supplementary material for: Safety and efficacy of bempedoic acid among patients with statin intolerance and those without: A meta-analysis and a systematic randomized controlled trial review
Source: PLoS One. 2024 Jan 26;19(1):e0297854. doi: 10.1371/journal.pone.0297854 (PMC10817114; doi:10.1371/journal.pone.0297854)
Supplement: S1 Table — (DOCX) [file pone.0297854.s004.docx]

**Table 1**Basic features of the included studies

| Author | Year | Study | Population | Number | Intervention | control | Outcome | Treatment duration | Age(years),mean ± SD | Female, n (%) |
| --- | --- | --- | --- | --- | --- | --- | --- | --- | --- | --- |
| S.E. Nissen | 2023 | RCT | patients who were unable or unwilling to take statins or were at high risk for cardiovascular disease | 13,970 | bempedoic acid | placebo | component composite of major adverse cardiovascular events | 40.6 months | 65.5±9.0 | 6740(48.2) |
| John Rubino | 2021 | RCT | patients with hypercholesterolemia | 58 | bempedoic acid | placebo | percent change in LDL-C | 2months | 60.14±10.44 | 36(62.1) |
| John Rubino | 2021 | RCT | patients with hypercholesterolemia | 63 | bempedoic acid,ezetimibe,atorvastat | placebo | percent change in LDL-C | 6weeks | 61.2±11.0 | 40（63.5） |
| Christie M Ballantyne | 2020 | RCT | patients with hypercholesterolemia and high CVD risk | 301 | bempedoic acid,ezetimibe | placebo | percentage change in LDL-C | 12weeks | 64.3±9.50 | 152(50.5) |
| Kausik K. Ray | 2019 | RCT | patients with atherosclerotic cardiovascular disease, heterozygous familial hypercholesterolemia, or both | 2230 | bempedoic acid | placebo | percentage change in LDL-C | 12 to 52 weeks | 66.1±8.9 | 602(27.0) |
| Ulrich Laufs | 2019 | RCT | patients with hypercholesterolemia and statin Intolerance | 345 | bempedoic acid | placebo | percent change in LDL-C | 24weeks | 65.2±9.5 | 194（56.2） |
| Narendra D. Lalwani | 2019 | RCT | patients with hypercholesterolemia | 64 | bempedoic acid | placebo | the LDL-C lowering efficacy | 4weeks | 58±9.3 | 31(48.4) |
| Anne C. Goldberg | 2019 | RCT | patients with atherosclerotic cardiovascular disease, heterozygous familial hypercholesterolemia, or both | 779 | bempedoic acid | placebo | percent change in LDL-C level | 52weeks | 64.3±8.8 | 283(36.3) |
| Christie M. Ballantyne | 2018 | RCT | patients with a history of statin intolerance | 269 | bempedoic acid | placebo | percent change in LDL-C. | 12weeks | 63.8±10.9 | 165(61.3) |
| Paul D. Thompson | 2016 | RCT | hypercholesterolemic patients | 348 | bempedoic acid | placebo | percent change in LDL-C | 12weeks | 59.9±9.6 | 265（79.1） |
| Christie M. Ballantyne | 2016 | RCT | hypercholesterolemic Patients | 133 | bempedoic acid | placebo | percent change in LDL-C | 12weeks | 57.3±9.7 | 79（59.4） |
| Paul D. Thompson | 2015 | RCT | hypercholesterolemia in patients with statin intolerance | 56 | bempedoic acid | placebo | percentage change in LDL-C | 8weeks | 62.6±6.4 | 28(50.0%) |
| Maria J. Gutierrez | 2014 | RCT | patients with type 2 diabetes mellitus and hypercholesterolemia | 60 | bempedoic acid | placebo | the lipid-altering effects | 4weeks | 55.7±8.5 | 23(38.3) |
| Christie M. Ballantyne | 2013 | RCT | patients with hypercholesterolemia | 177 | bempedoic acid | placebo | changes in LDL-C | 12weeks | 57.7±9.5 | 79（44.6） |
| NCT03531905 | 2020 | RCT | patients with type 2 diabetes (T2D) and elevated LDL-C | 242 | bempedoic acid | placebo | LDL-C lowering | 12weeks | 61.4±8.4 | 117(48.3) |
| NCT02178098 | 2023 | RCT | participants with hypercholesterolemia and hypertension | 143 | bempedoic acid | placebo | the efficacy and safety of ETC-1002 | 6weeks | 55.6±8.4 | 61(42.7) |
