# Supplementary material for: Safety and efficacy of bempedoic acid among patients with statin intolerance and those without: A meta-analysis and a systematic randomized controlled trial review
Source: PLoS One. 2024 Jan 26;19(1):e0297854. doi: 10.1371/journal.pone.0297854 (PMC10817114; doi:10.1371/journal.pone.0297854)
Supplement: Attachment — Submitted filename: Response to Reviewers.doc [file pone.0297854.s006.doc]

**Response to Reviewers**

December 18, 2023

Dear Editor,

We would like to thank you and all reviewers for your insightful comments concerning the revisions of our manuscript entitled “*Safety and efficacy of bempedoic acid among patients with statin intolerance and those without: A meta-analysis and a systematic randomized controlled trial review”*. We have carefully reviewed the reviewers’ comments, and we have addressed all their questions/critiques/concerns point-by- point as below. All changes made to the text were tracked so that they may be easily identified. We believe that our manuscript has been significantly improved, and we hope it is now suitable for publication.

If you have any problems with our manuscript, please do not hesitate to contact us.

**Journal Requirements:**

When submitting your revision, we need you to address these additional requirements.

1. Please ensure that your manuscript meets PLOS ONE's style requirements, including those for file naming. The PLOS ONE style templates can be found at
   <https://journals.plos.org/plosone/s/file?id=wjVg/PLOSOne_formatting_sample_main_body.pdf> and
   <https://journals.plos.org/plosone/s/file?id=ba62/PLOSOne_formatting_sample_title_authors_affiliations.pdf>

Reply: Thank you, we have adjusted the format according to your suggestion

2. Did you know that depositing data in a repository is associated with up to a 25% citation advantage (<https://doi.org/10.1371/journal.pone.0230416>)? If you’ve not already done so, consider depositing your raw data in a repository to ensure your work is read, appreciated and cited by the largest possible audience. You’ll also earn an Accessible Data icon on your published paper if you deposit your data in any participating repository ([https://plos.org/open-science/open-data/#accessible-data](https://plos.org/open-science/open-data/#accessible-data)).

Reply: Thank you for your valuable advice, we will not consider this for the time being

3. PLOS requires an ORCID iD for the corresponding author in Editorial Manager on papers submitted after December 6th, 2016. Please ensure that you have an ORCID iD and that it is validated in Editorial Manager. To do this, go to ‘Update my Information’ (in the upper left-hand corner of the main menu), and click on the Fetch/Validate link next to the ORCID field. This will take you to the ORCID site and allow you to create a new iD or authenticate a pre-existing iD in Editorial Manager. Please see the following video for instructions on linking an ORCID iD to your Editorial Manager account: <https://www.youtube.com/watch?v=_xcclfuvtxQ>

Reply: Thank you, we have followed your suggestion. Thank you again
4. Please review your reference list to ensure that it is complete and correct. If you have cited papers that have been retracted, please include the rationale for doing so in the manuscript text, or remove these references and replace them with relevant current references. Any changes to the reference list should be mentioned in the rebuttal letter that accompanies your revised manuscript. If you need to cite a retracted article, indicate the article’s retracted status in the References list and also include a citation and full reference for the retraction notice.

Reply: Thank you, we have followed your suggestion. Thank you again

**Additional Editor Comments:**

Authors have produced a scientifically sound paper that can be accepted after minor revisions to address comments of the reviewers.

Reply: Thank you for your valuable advice. We have carefully reviewed the reviewers’ comments, and we have addressed all their questions/critiques/concerns point-by- point as below. All changes made to the text were tracked so that they may be easily identified. We believe that our manuscript has been significantly improved, and we hope it is now suitable for publication.

Reviewers' comments:
Reviewer's Responses to Questions

**Comments to the Author**

1. Is the manuscript technically sound, and do the data support the conclusions?

The manuscript must describe a technically sound piece of scientific research with data that supports the conclusions. Experiments must have been conducted rigorously, with appropriate controls, replication, and sample sizes. The conclusions must be drawn appropriately based on the data presented.

Reviewer #1: Yes

Reviewer #2: Yes

Reply: Thank you for your affirmation, thank you

2. Has the statistical analysis been performed appropriately and rigorously?

Reviewer #1: Yes

Reviewer #2: Yes

Reply: Thank you for your affirmation, thank you

3. Have the authors made all data underlying the findings in their manuscript fully available?

The [PLOS Data policy](http://www.plosone.org/static/policies.action#sharing) requires authors to make all data underlying the findings described in their manuscript fully available without restriction, with rare exception (please refer to the Data Availability Statement in the manuscript PDF file). The data should be provided as part of the manuscript or its supporting information, or deposited to a public repository. For example, in addition to summary statistics, the data points behind means, medians and variance measures should be available. If there are restrictions on publicly sharing data—e.g. participant privacy or use of data from a third party—those must be specified.

Reviewer #1: Yes

Reviewer #2: Yes

Reply: Thank you for your affirmation, thank you

4. Is the manuscript presented in an intelligible fashion and written in standard English?

PLOS ONE does not copyedit accepted manuscripts, so the language in submitted articles must be clear, correct, and unambiguous. Any typographical or grammatical errors should be corrected at revision, so please note any specific errors here.

Reviewer #1: Yes

Reviewer #2: Yes

Reply: Thank you for your affirmation, thank you

5. Review Comments to the Author

Please use the space provided to explain your answers to the questions above. You may also include additional comments for the author, including concerns about dual publication, research ethics, or publication ethics. (Please upload your review as an attachment if it exceeds 20,000 characters)

Reviewer #1: The metanalysis although showed that Bempedoic acid is efficacious in reducing LDL cholesterol and no increase in the muscle associated occurrences there is drawback of drug discontinuation due to adverse effects,

Authors should highlight the major adverse effects that lead to discontinuation of treatment.

Reply: Thank you for your valuable advice. We have highlighted the major adverse effects that lead to discontinuation of treatment in the discussion section.

Authors should analyze the NET clinical benefit balancing the advantage of absence of muscle associated symptoms and downside of more adverse effects.

Reply: We agree. When evaluating the clinical benefit of a medication like bempedoic acid, it is crucial for authors to analyze the net clinical benefit by weighing the advantage of the absence of muscle-associated symptoms against the potential downside of experiencing more adverse effects.By highlighting both the advantages and potential adverse effects, authors can provide a balanced assessment of the medication's clinical benefit.We have revised the discussion section as suggested.

Reviewer #2: Good meta analysis on Bempedoic acid - a currently underutilized lipid lowering therapy.
Reply: Thank you for your affirmation, thank you
Some minor observations are mainly on spelling and grammatical errors:
Line 129 should read "except" rather than "expect"
Line 236 should read "subgroup analyses report"
Line 243 should read "compared to" instead of "than"
Line 254 should read "requires"

Reply: Thanks for the reminder. We have revised the manuscript as suggested.
